# Development of a competency framework for postgraduate training in obstetrics and gynaecology using a Delphi study

**DOI:** 10.5116/ijme.679e.0509

**Published:** 2025-02-24

**Authors:** Ellen Allaert, Marieke Robbrecht, Tjalina Hamerlynck, Steven Weyers

**Affiliations:** 1 Ghent University, Faculty of Medicine and Health Sciences, and Women's Clinic, Ghent University Hospital, Belgium; 2 Ghent University, Faculty of Medicine and Health Sciences, and Department of Paediatrics, Ghent University Hospital, Belgium

**Keywords:** Gynaecology, obstetrics, competency framework, post-graduate training, Delphi methodology

## Abstract

**Objectives:**

The aim of this study was to create a new integrated competency framework for the postgraduate training in obstetrics and gynaecology and to reach consensus through a Delphi study.

**Methods:**

Using the Canadian Medical Education Directives for Specialists (CanMEDS) framework as a basis, three existing frameworks were merged by screening for keywords. Subsequently, consensus on the unified framework was reached through a Delphi study: a group of 18 Belgian experts was asked for their opinions on the competencies through three successive questionnaires.

**Results:**

In the first round, one of the in total 91 competencies was deemed irrelevant. In the second round, the competencies were reviewed for content and formulation, after which consensus was not reached on 15 competencies. These 15 competencies were adjusted as needed based on comments collected during the first two rounds. The adjusted competencies were then sent back to the experts in the third round, resulting in a final consensus on all 91 competencies. However, the comments indicated that several competencies were considered broad or vague, casting doubt on their practical applicability.

**Conclusions:**

Through a Delphi study, consensus was reached on a newly composed competency framework. Such a holistic competency framework can form the basis of a curriculum reform in the postgraduate training in obstetrics and gynaecology within Belgium, but also in a more international context. Further research is needed to develop an assessment tool to implement these competencies in practice.

## Introduction

Since the turn of the century, Competency-Based Medical Education (CBME) has found its implementation in residency training.^1^ Traditionally, medical education is focused on time-based education, where the focus lies on the process of learning, and on the exposure to learning opportunities for specified periods of time.^2^^-^^4^ Competency-based education emphasises on the product of learning: the outcome drives the educational process. How the outcome is reached is secondary.^1^^-^^3^^, ^^5^^, ^^6^

Although Competency-Based Education (CBE) has been implemented in multiple professional environments for many years (e.g. NASA, the military), it is only since the last decades that the benefits of CBE in medicine became clear.^7^ CBME enhances transparency in medical education, empowers students, enables comparison of curricula in different countries, and improves patient safety.^1^^, ^^7^^-^^9^ Moreover, CBME addresses the needs for evidence-based, cost-effective, patient-centred health care.^10^ Many countries, such as the United States, Canada, Australia, the Netherlands and the United Kingdom, already use a competency-based national educational program for residency training.^1^^, ^^4^^, ^^11^

Over the years, specific competency frameworks have been developed for postgraduate training in obstetrics and gynaecology, such as the Objectives of Training in the Specialty of Obstetrics and Gynecology^12^ in Canada and the Dutch national training program for gynaecology and obstetrics (‘LOGO’).^13^ In Belgium no uniform framework exists for postgraduate training in obstetrics and gynaecology. The general CanMEDS framework has been adopted in the curriculum of several Belgian universities for undergraduate training.^11^^,^^14^ A framework defined for MMSM (Master of Medicine in Specialist Medicine) is based on the CanMEDS competency framework, utilizing four CanMEDS roles as the foundation for the rest of the framework. This framework is used for feedback and evaluation during workplace-based learning in all specialistic medicine disciplines. The original CanMEDS framework is not included in postgraduate training in Belgium. The competency framework of the European Union of Medical Specialists (UEMS), more specific the European Board and College of Obstetrics and Gynaecology (EBCOG) and the European Network of Trainees in Obstetrics and Gynaecology (ENTOG), provides an overview of all the knowledge and technical skills that a trainee in obstetrics and gynaecology should master upon certification.^15^ It is used as a guideline for training and assessmentbut is not officially incorporated in the postgraduate curriculum. The absence of a uniform framework complicates the evaluation of residents from different institutions and hinders the final assessment of medical competence for certification. At an international level, the variation hampers the evaluation of a resident's level of proficiency and therefore complicates exchange of residents and knowledge.

Given the increasing importance of high-quality care and the growing internationalisation of medicine, it is crucial that the postgraduate training in obstetrics and gynaecology in Belgium is standardised. A uniform competency framework can also serve as a basis within a more international context, simplifying the process for international equivalence of diplomas. In this study, the goal was to develop a new competency framework, based on the merging of the three existing frameworks. As a Delphi study is typically used for defining competencies and curriculum development, this study method was chosen to subsequently achieve consensus on the newly merged competency framework.^33^

This new competency framework will be used for the SBO Scaffold project, aiming to design an evidence-based ePortfolio that supports healthcare students in their competence development at the workplace.^16^ Considering the international background of the used frameworks, this could be an example for other countries to reform their curriculum or assessment tools.

## Methods

### Generating the competency framework

In order to construct a new competency framework, three pre-existing frameworks were used: The CanMEDS roles as defined by The Royal College of Physicians and Surgeons of Canada^12^, the European Training Requirements in Obstetrics and Gynaecology as defined by the UEMS ^15^ and the competencies as defined by the MMSM.^17^

The CanMEDS competencies were selected as a foundation for the new framework because these have already been validated and implemented in the postgraduate medical training in numerous countries.^11^^, ^^18^^-^^21^ The integration of the UEMS framework was essential because this framework was recently developed to improve the European standards of training.^22^ Since the competencies as defined by MMSM are already used by several Belgian universities, these could not be left out.

First, all the CanMEDS competencies were listed out in a Microsoft Excel file by the main investigator (EA). Subsequently, the UEMS competencies were linked to the matching CanMEDS roles and consecutively to the matching key competencies and enabling competencies. During the process, this step was reviewed by a specialised research group associated with the SBO Scaffold project. All steps were repeated for the competencies of the MMSM framework.

**Figure 1:**
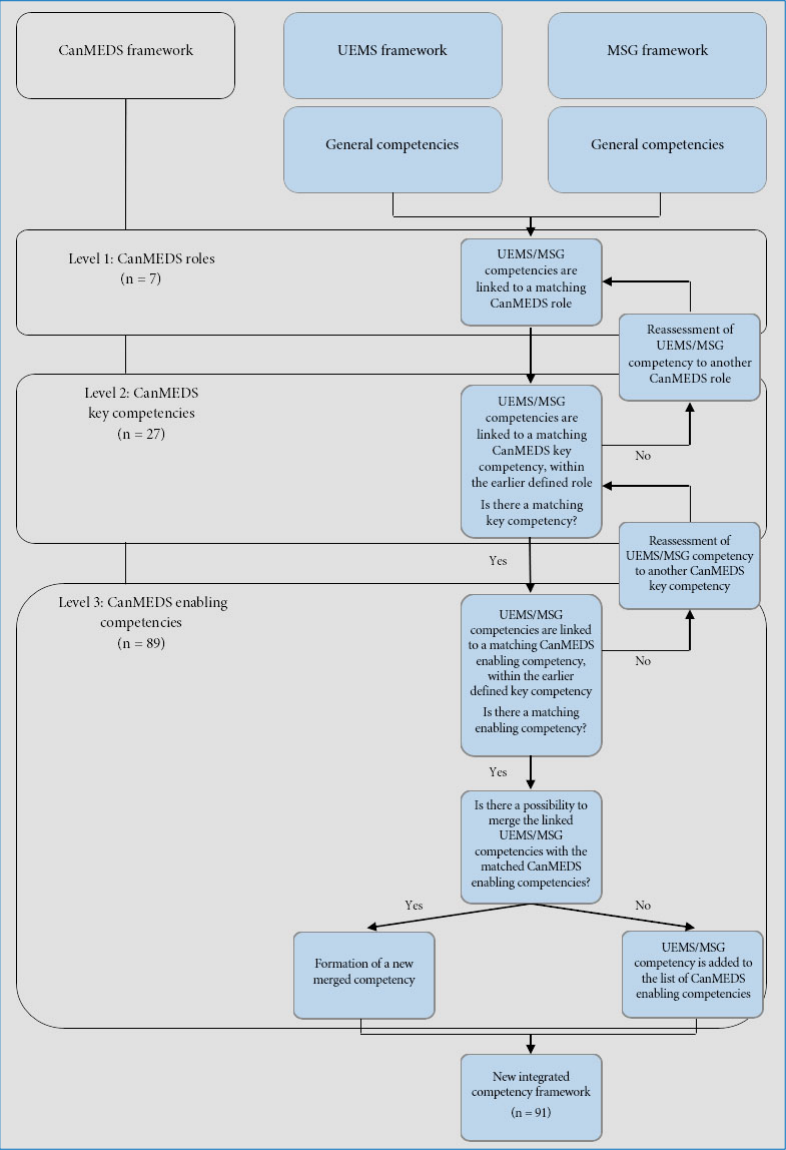
Flowchart of the process to generate the competency framework

After linking all UEMS and MMSM competencies to the CanMEDS enabling competencies, the entire list was checked for gaps and overlap. Where possible, overlapping competencies were merged based on contained keywords. If combining competencies was not possible without changing the essence, these UEMS or MMSM competencies were added to the list of CanMEDS competencies. This step was again reviewed by the research group. In total 33 UEMS competencies and 33 MMSM competencies were merged with the 89 CanMEDS enabling competencies to create a new framework of 91 competencies. A flowchart of these steps can be found in Figure 1.

### Study design

A Delphi methodology was used to reach consensus on the developed competency framework through an online questionnaire. Classically, a Delphi procedure consists of several rounds, during which expert opinions are assimilated using controlled feedback, ultimately leading to a group consensus.^11^^, ^^24^^-^^28^ It has a quasi-anonymous design: the identity of the respondents is known to the main investigator, but their answers and opinions remain strictly anonymous to the other respondents.^26^^, ^^29^

For this study, an e-Delphi was used, which is a web-based technique using online questionnaires. An online format facilitates participation from diverse geographical locations, is cost-effective and allows respondents to fill in the questionnaire at a moment that suits their personal agenda.^25^^, ^^30^

### Design questionnaire

During the first Delphi round, participants were asked to assess all competencies for relevance by using a 6-point Likert scale (1 = not at all relevant to 6 = very relevant).^28^ In addition to the Likert scale, participants were able to give comments or specify their chosen answer during every round. The second round contained the list of all competencies, complemented by the level of consensus reached for each competency and the qualitative comments collected in the first round.^11^^, ^^29^ During this round, participants were asked to review the formulation and content and indicate whether they agreed with leaving the competency as it currently stands or not. All competencies without consensus regarding formulation or content were retained and, if necessary, adjusted, based on the feedback of the experts. This process was done in consultation with the research group and two appointed gynaecologists (SW, TH) involved in training residents in obstetrics and gynaecology at the Ghent University Hospital. All adjusted competencies were submitted to the participants during the third round, to determine whether they agreed with the implemented adjustments or not. This in order to reach total group consensus.^27^

The survey was not piloted, indication of time required to complete the survey, reliability and feasibility of the study was ensured by a similar study conducted by a researcher in the research group.^23^

**Figure 2:**
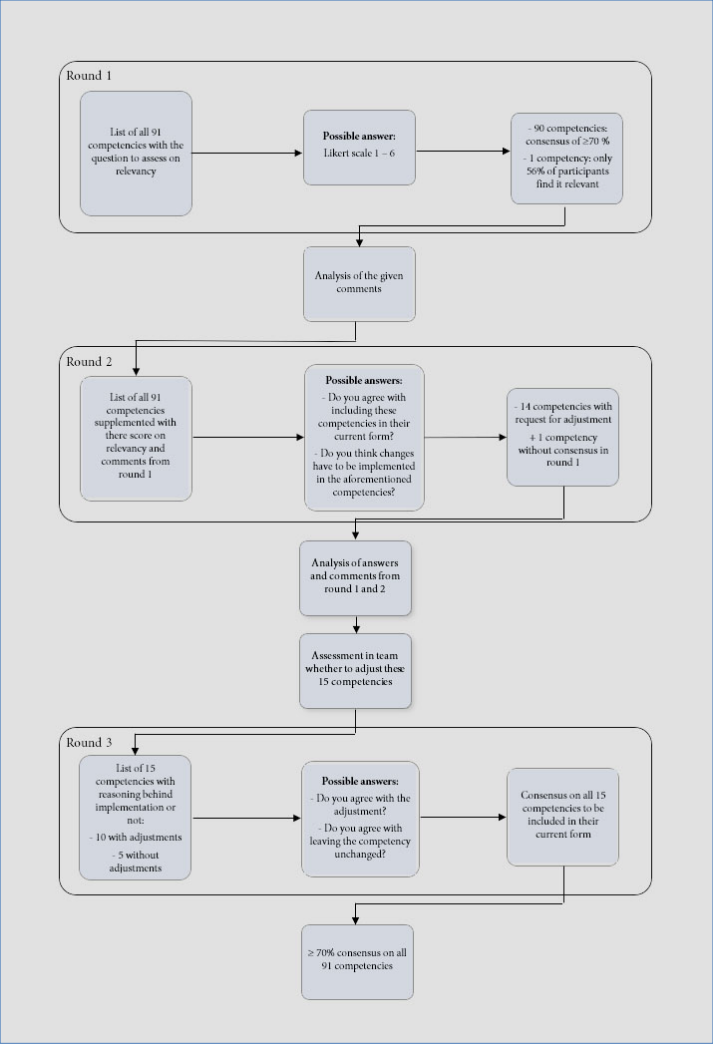
Flowchart of the Delphi study

### Selection of experts

Participants of a Delphi study are considered to be experts in their field with knowledge about the area of research.^24^^,^^28^^,^^29^ Choosing appropriate experts with knowledge and interest in the topic has a direct positive correlation to the quality of the results, the risk of bias and the content validity of the study.^26^^, ^^29^^, ^^31^

For this study, five groups of experts were included: 1) the recognition committee for obstetrician-gynaecologists in Flanders, 2) supervisors of residents-in-training for obstetrics and gynaecology affiliated with a Belgian University, 3) experts in medical education, involved in competency-based education, 4) recently (2020) recognised obstetrician-gynaecologists, 5) a member of the EBCOG.

Ethical approval was obtained from the Ethics Committee of the Ghent University Hospital. Informed consent was obtained from all participants.

### Data collection

An email request for participation was sent to all eligible experts. All respondents subsequently received a personal link by email to the online questionnaire of round 1, created with Qualtrics. The same steps were followed for rounds 2 and 3. There was a period of one and a half months between each round. An overview of what personal link belonged to which participant was retained, only accessible for the main investigator. Data was collected between September 2021 and January 2022 and subsequently stored on a secured Ghent University server. All results collected in Qualtrics were transferred round by round to an Excel document where data analyses could be performed. The survey was conducted in English to make sure both Flemish and French speaking experts could participate and to decrease the risk of translation bias, since the CanMEDS framework is in English. In addition, an English framework facilitates further investigation in an international context. To enhance response rates, participants who had not (fully) filled in the questionnaire were individually sent one or two reminder emails.^25^^, ^^26^^, ^^29^

### Data analysis

After collection, all analyses were performed in Microsoft Excel. For the first round, percentages of relevance were calculated. Using the 6-point Likert scale, competencies with a score from 4 to 6 were rated as relevant. Consensus was reached when at least 70% of the participants gave a score of 4 or higher. This threshold of 70% has been repeatedly used in previous Delphi studies.^26^^, ^^30^^, ^^32^^, ^^33^ During the second round, competencies were included when at least 70% of experts agreed not to change the competency. All competencies that did not reach consensus were collected and analysed based on the qualitative comments received. Qualitative comments were analysed and subsequently categorised into themes through inductive content analysis.^34^ Based on the analysis of the qualitative comments, the third round of the questionnaire was constructed.^25^^, ^^28^ Competencies with a 70% consensus to accept the adjustments in round three were included in the final competency framework.

## Results

### Demographics

A group of 122 experts was approached, of which 25 responded. After the first round, 17 out of 25 participants completed the questionnaire and 2 participants started but did not finish (73%). For the second round, 19 requests were sent, and 16 questionnaires were finished, of which 1 incomplete (84%). A total of 15 out of 16 experts (93%) participating in round two, completed the third round. The demographics of all participants who completed at least one round (n = 18) are listed in Table 1.

### Study progress

An overview of the flow of the Delphi study can be found in Figure 2.

#### Round 1

The new framework, consisting of 91 competencies, was sent to the participants to assess for relevance. A consensus of 100% was reached for 40 out of 91 competencies. A consensus of >70% was reached for 50 competencies, only one competency did not reach the 70% consensus threshold.

A total of 38 qualitative comments were given, these could be clustered into 6 categories: general remarks on the study (n=4), comments on a clerical error (n=6), suggestions to adjust the phrasing (n=11), questions about practical feasibility (n=5), suggestions to merge or split competencies (n=10) and additional information about their own scoring (n=2).

#### Round 2

During round 2, all 91 competencies were supplemented with their scores from round 1 and their specific comments. Participants were asked to judge if a competency should be adjusted or not, based on the provided feedback.

**Table 1:** Demographics, 18 respondents, online questionnaire, Belgium, 2021-2022

Variable		N
Age (years)	31 - 35	5
	36 - 40	1
	46 - 50	4
	51 - 55	1
	56 - 60	1
	61 - 65	4
	66 - 70	1
	>70	1
Functions*	Recently graduated gynaecologist or obstetrician (2020)	3
	Member of the recognition committee for ob/gyns	4
	Member of the study programme committee	6
	Supervisor of ob/gyns in training affiliated with a Belgian University	8
	Member of the ob/gyn section of UEMS: EBCOG	1
University†	Leuven	5
	Antwerp	1
	Ghent	8
	Brussels	2
Supervisedresidents per year†	3	2
	5	3
	6 or more	6
	Not applicable	7

EBCOG = European Board and College of Obstetrics and Gynaecology, UEMS = European Union of Medical Specialists * Some participants had multiple functions, making the total amount greater than 18 † This information was not available for all 18 participants

A consensus of 100% was reached for 29 competencies to retain the original formulation. No consensus was reached for 14 competencies, meaning there was a request for adjustment.

The remaining 47 competencies received at least a 70% consensus. The competency that did not reach consensus on relevance during the first round, did also not reach consensus to be excluded from the competency framework during the second round. Therefore, this competency was added to the list of competencies that needed adjustment. In total 15 competencies were selected to be adjusted.

There were 51 qualitative comments in the second round. All comments were analysed within the research group, but only those referring to the competencies without consensus (n=15) were taken into account. The remaining comments (n=36) were judged to be not significantly important to change a competency that already reached consensus.

The 15 relevant comments could be divided into 4 categories: suggestions how to adjust the competency (n=8), doubt about relevance for the trainee (despite being scored as relevant during the first round) (n=2), ideas to apply the competency into practice (n=2) and additional information about their own scoring (n=3). Based on these comments and in agreement with the research group, 10 competencies were adjusted, and 5 competencies were left unchanged. All 15 competencies without consensus can be found in Table 2, together with the adjustments (if carried out) and the reasoning behind implementation or non-implementation of the adjustments.

#### Round 3

During the third and last round, all 15 competencies reached consensus, of which 7 reached a 100% consensus. One adjusted competency, namely “Promote a culture that recognises, supports, and responds effectively to colleagues in need by facilitating the process to help”, received 4 comments saying that the implemented adjustment was unnecessary. Nevertheless, a consensus of 73,3% was reached, therefore the adjustment was retained. The final version of the competency framework is listed in Table 3.

## Discussion

A new competency framework for postgraduate training in obstetrics and gynaecology was merged based on three pre-existing frameworks and reached consensus through a Delphi study including Belgian experts.

When using a Delphi study for qualitative research, it is important that a correct study design is used to increase content validity. During this Delphi study only 15 participants completed the entire study, although the researchers aimed to include 30 participants. Since a Delphi study is very time consuming, it is possible that only experts with special interests in the topic or experts with more free time than average participated, increasing the risk of bias. However, all five expert groups as listed prior to the onset of the study were represented by the study population, which reflects a representative and qualitative group.^31^ A response rate of at least 70% was reached for every round and the level of consensus was defined prior to the study onset. We can state that the content validity of this Delphi study is adequate, based on the research design that was used.

Based on this study, we hoped to compile a more integrated competency framework that could form the basis of a standardised national training program in postgraduate training in obstetrics and gynaecology. By integrating both general and discipline-specific competencies within one competency framework, trainees can take more control over their training and evaluation moments can be better substantiated. Thanks to the predetermined competencies, it is clearer for trainees and supervisors what is expected of them.^1^

A similar study was conducted within the paediatric discipline, where consensus was reached on a very similar competency framework.^23^ This confirms that a competency framework, as compiled in this study, is also supported by experts within their field in other disciplines. Moreover, this is a confirmation that a holistic competency framework like this could potentially serve as a basis for curriculum reform of all specialist training programs in Belgium.

**Table 2:** Competencies without consensus, with adjustments (if carried out)

No	Competency before Delphi study	Adjusted competency after Delphi study	Argumentation for adjustment
1	Have and apply knowledge about embryology, anatomy and the physiology of the female genital organs and breasts and understand bio-psychosocial aspects of obstetrical and gynaecological conditions as listed in addendum* (ADDENDUM: OBSTETRICAL AND GYNAECOLOGICAL EXPERTISE)	Have and apply knowledge about embryology, anatomy, genetics and the physiology of the female genital organs and breasts and understand bio-psychosocial aspects of obstetrical and gynaecological conditions as listed in addendum* (ADDENDUM: OBSTETRICAL AND GYNAECOLOGICAL EXPERTISE)	Genetics is also important to have knowledge about
2	Prioritize issues to be addressed in a patient encounter	Prioritize issues to be addressed in a patient encounter, based on urgency and further planning	Relevant, but very general statement. Attempt to make this competency more concrete.
3	Establish a patient-centred management plan	Establish a concrete patient-centred management plan, based on the postulated goals	Very similar to a previous competency. Difference is that the former competency aims at defining goals, while this aims at the way these goals can be reached.
4	Perform the gynaecologic and obstetric skills as listed in addendum, in a skilful and safe manner, adapting to unanticipated findings or changing clinical circumstances (ADDENDUM: PRACTICAL SKILLS )	Left unchanged	Comments on the list in addendum, which is not yet completed. No comments on formulation of the competency.
5	Implement a patient-centred care plan that supports ongoing care, follow-up on investigations, response to treatment, and further consultation	Left unchanged	Comment that there is overlap with former competencies:Other competencies cover the process of obtaining a treatment goal and establishing a plan.This competency covers the implementation of the care plan
6	Deliver the highest quality of care, including the adoption of strategies that contribute to the promotion of patient safety, and address human and system factors	Deliver the highest evidence based quality of care, including the adoption of strategies that contribute to the promotion of patient safety, and address human and system factors	Defines more specifically what the highest quality of care means
7	Apply the science of quality improvement to contribute to improving systems of patient care	Contribute to the improvement of systems of patient care, by applying the science of quality improvement	Comment that there is overlap with a former competency: Focus on the improvement of systems of patient care, as a leader.
8	Allocate health care resources for optimal patient care	Allocate health care resources, such as equipment, human and financial resources, for optimal patient care	It goes further than only financial resources
9	Manage health care in a socially responsible way and demonstrate leadership skills to enhance health care	Left unchanged	Comment that there is overlap with a former competency, but unclear which competency is meant. Presumably a competency regarding cost-appropriate care was meant. Socially responsible health care is much broader than cost-appropriate care
10	Manage stressful situations with effective responses to challenge, complexity and stress in gynaecology and obstetrics	Manage and lead stressful situations with effective responses to challenge, complexity and stress in gynaecology and obstetrics	Focus on the management of stressful situations as a leader
11	Work with a community or population to identify the determinants of health that affect them	Work with a community or population to identify the determinants of health that affect them (If applicable)	No consensus regarding relevancy for all ObGyns
12	Improve clinical practice by applying a process of continuous quality improvement to disease prevention, health promotion, and health surveillance activities	Left unchanged	Overlap with other competencies on quality of care. This competency focuses on continuous quality improvement in three areas which are not yet covered in other competencies
13	Develop, implement, monitor, and revise a personal learning plan to enhance professional practice	Left unchanged	Comment that another competency covers this statement too.This competency focuses on the development of a personal learning plan and not on the method of learning
14	Recognize and respond to unprofessional and unethical behaviours in physicians and other colleagues in the health care professions	Recognize and respond to unprofessional and unethical behaviours in physicians and other colleagues in the health care professions, according to the principles of effective feedback	Describes how someone is expected to respond
15	Promote a culture that recognizes, supports, and responds effectively to colleagues in need	Promote a culture that recognizes, supports, and responds effectively to colleagues in need by facilitating the process to help	Defines more specifically what "responds effectively" means

**Table 3:** Final competency framework

Role 1. Medical expert		
	Key competency 1: Practise medicine within their defined scope of practice and expertise	
		Have and apply knowledge about embryology, anatomy, genetics and the physiology of the female genital organs and breasts and understand bio-psychosocial aspects of obstetrical and gynaecological conditions as listed in addendum* (ADDENDUM: OBSTETRICAL AND GYNAECOLOGICAL EXPERTISE)
		Perform appropriately timed clinical assessments with adequate responsiveness to situations where the wellbeing of the patient is endangered or compromised, and present recommendations in an organized manner
		Carry out professional duties in the face of multiple, competing demands
		Recognize and respond to the complexity, uncertainty, and ambiguity inherent in medical practice
	Key competency 2: Perform a patient-centred clinical assessment and establish a management plan	
		Prioritize issues to be addressed in a patient encounter, based on urgency and further planning
		Elicit a patient and family history including social issues, perform a physical exam, select appropriate investigations, and interpret their results for the purpose of (differential) diagnosis and management, disease prevention, and health promotion
		Establish goals of care in collaboration with patients and their families, which may include slowing disease progression, treating symptoms, achieving cure, improving function, and palliation
		Establish a concrete patient-centred management plan, based on the postulated goals
	Key competency 3: Plan and perform procedures and therapies for the purpose of assessment and/or management	
		Order the appropriate investigations for gynaecologic and obstetric assessment; interpret their results for the purpose of formulating an appropriate (differential) diagnosis; determine the most appropriate therapies or preventive interventions including the safe prescription of common drugs; all in an evidence-based manner
		Obtain and document informed consent, explaining the risks and benefits of, and the rationale for, a proposed procedure or therapy
		Prioritize a procedure or therapy, taking into account clinical urgency and available resources
		Perform the gynaecologic and obstetric skills as listed in addendum, in a skilful and safe manner, adapting to unanticipated findings or changing clinical circumstances (ADDENDUM: PRACTICAL SKILLS)
	Key competency 4: Establish plans for ongoing care and, when appropriate, timely consultation	
		Implement a patient-centred care plan that supports ongoing care, follow-up on investigations, response to treatment, and further consultation
	Key competency 5: Actively contribute, as an individual and as a member of a team providing care, to the continuous improvement of health care quality and patient safety	
		Recognize and respond to harm from health care delivery, including patient safety incidents
		Identify the limits of one's own competency and act within them by asking for help when needed
		Deliver the highest evidence-based quality of care, including the adoption of strategies that contribute to the promotion of patient safety, and address human and system factors
Role 2. Communicator		
	Key competency 1: Establish professional therapeutic relationships with patients and their families	
		Communicate using active listening and a patient-centred approach that encourages patient trust and autonomy and is characterized by empathy, respect, and compassion
		Optimize the physical environment for patient comfort, dignity, privacy, engagement, and safety
		Recognize when the values, biases, or perspectives of patients, physicians, or other health care professionals may have an impact on the quality of care, and modify the approach to the patient accordingly
		Respond to a patient’s non-verbal behaviours to enhance communication
		Manage disagreements and emotionally charged conversations
		Adapt to the unique needs and preferences of each patient and to his or her clinical condition and circumstances
	Key competency 2: Elicit and synthesize accurate and relevant information, incorporating the perspectives of patients and their families	
		Use patient-centred interviewing skills to effectively gather relevant biomedical and psychosocial information
		Provide a clear structure for and manage the flow of an entire patient encounter
		Seek and synthesize relevant information from other sources, including the patient’s family, with the patient’s consent
	Key competency 3: Share health care information and plans with patients and their families	
		Share information and explanations that are clear, accurate, and timely, while checking for patient and family understanding
		Disclose harmful patient safety incidents to patients and their families accurately and appropriately
	Key competency 4: Engage patients and their families in developing plans that reflect the patient’s health care needs and goals	
		Facilitate discussions with patients and their families in a way that is respectful, non-judgmental, and culturally safe
		Assist patients and their families to identify, access, and make use of information and communication technologies to support their care and manage their health, ensuring patient empowerment
		Use communication skills and strategies that help patients and their families make informed decisions regarding their health, facilitating the balance between evidence-based recommendations and patient's preferences
	Key competency 5: Document and share written and electronic information about the medical encounter to optimize clinical decision-making, patient safety, confidentiality, and privacy	
		Document clinical encounters in an accurate, complete, timely, and accessible manner, in compliance with regulatory and legal requirements
		Communicate effectively using a written health record, electronic medical record, or other digital technology
		Share information with patients and others in a manner that respects patient privacy and confidentiality and enhances understanding
Role 3. Collaborator		
	Key competency 1: Work effectively with physicians and other colleagues in the health care professions	
		Establish and maintain positive relationships with physicians and other colleagues in the health care professions, by focussing on team performance and developing effective communication, to support relationship-centred collaborative care and contribute to a constructive working environment
		Negotiate overlapping and shared responsibilities with physicians and other colleagues in the health care professions in episodic and ongoing care
		Engage in respectful shared decision-making with physicians and other colleagues in the health care professions, recognizing and relying on the expertise of others
	Key competency 2: Work with physicians and other colleagues in the health care professions to promote understanding, manage differences, and resolve conflicts	
		Show respect toward collaborators
		Implement strategies to promote understanding, manage differences, and resolve conflicts in a manner that supports a collaborative culture
	Key competency 3: Hand over the care of a patient to another health care professional to facilitate continuity of safe patient care	
		Determine when care should be transferred to another physician or health care professional
		Demonstrate safe handover, referral and discharge planning of care; using both verbal and written communication during a patient transition to a different health care professional, setting or stage of care
Role 4. Leader		
	Key competency 1: Contribute to the improvement of health care delivery in teams, organizations, and systems	
		Contribute to the improvement of systems of patient care, by applying the science of quality improvement
		Contribute to the organisation of health care within their own facility
		Contribute to a culture that promotes patient safety
		Analyze patient safety incidents to enhance systems of care
		Use health informatics to improve the quality of patient care, optimize patient safety and maintenance of own expertise
		Contribute to the progress of health care by facilitating the implementation of innovation
	Key competency 2: Engage in the stewardship of health care resources	
		Allocate health care resources, such as equipment, human and financial resources, for optimal patient care
		Apply evidence and management processes to achieve cost-appropriate care
	Key competency 3: Demonstrate leadership in professional practice	
		Manage health care in a socially responsible way and demonstrate leadership skills to enhance health care
		Manage and lead stressful situations with effective responses to challenge, complexity and stress in gynaecology and obstetrics
		Facilitate change in health care to enhance services and outcomes
	Key competency 4: Manage career planning, finances, and health human resources in a practice	
		Set priorities and manage time, by working efficiently and organised, to integrate practice and personal life
		Manage a career and a practice
		Implement processes to ensure personal practice improvement
Role 5. Health advocate		
	Key competency 1: Respond to an individual patient’s health needs by advocating with the patient within and beyond the clinical environment	
		Work with patients to address determinants of health that affect them and their access to needed health services or resources
		Work with patients and their families to increase opportunities to adopt healthy behaviours
		Incorporate disease prevention, health promotion, and health surveillance into interactions with individual patients
	Key competency 2: Respond to the needs of the communities or populations they serve by advocating with them for system-level change in a socially accountable manner	
		Work with a community or population to identify the determinants of health that affect them (If applicable)
		Improve clinical practice by applying a process of continuous quality improvement to disease prevention, health promotion, and health surveillance activities
		Contribute to a process to improve health in the community or population they serve
Role 6. Scholar		
	Key competency 1: Engage in the continuous enhancement of their professional activities through ongoing learning	
		Develop, implement, monitor, and revise a personal learning plan to enhance professional practice
		Identify opportunities for learning and improvement by regularly seeking and accepting feedback and by reflecting on and assessing their performance using various internal and external data sources
		Engage in collaborative learning to continuously improve personal practice and contribute to collective improvements in practice
	Key competency 2: Teach students, residents, the public, and other health care professionals	
		Be a good role-model and recognize the influence of role-modelling and the impact of the formal, informal, and hidden curriculum on learners
		Promote a safe learning environment
		Ensure patient safety is maintained when learners are involved
		Plan and deliver a learning activity to students, colleagues and other healthcare professionals
		Provide adequate feedback to enhance learning and performance
		Assess and evaluate learners, teachers, and programs in an educationally appropriate manner
	Key competency 3: Integrate best available evidence into practice	
		Recognize practice uncertainty and knowledge gaps in clinical and other professional encounters and generate focused questions that address them
		Identify, select, and navigate pre-appraised resources
		Critically evaluate the integrity, reliability, and applicability of health-related research and literature
		Integrate evidence into decision-making in their practice
	Key competency 4: Contribute to the creation and dissemination of knowledge and practices applicable to health	
		Demonstrate an understanding of the scientific principles of research and scholarly inquiry and the role of research evidence in health care
		Identify ethical principles for research and incorporate them into obtaining informed consent, considering potential harms and benefits, and considering vulnerable populations
		Contribute to the progress of health care via the work of a research program (critical literature review, data collection and analysis, reporting research results)
		Pose questions amenable to scholarly inquiry and select appropriate methods to address them
		Summarize and communicate to professional and lay audiences, including patients and their families, the findings of relevant research and scholarly inquiry
Role 7. Professional		
	Key competency 1: Demonstrate a commitment to patients by applying best practices and adhering to high ethical standards	
		Exhibit appropriate professional behaviours and relationships in all aspects of practice, demonstrating honesty, integrity, humility, commitment, compassion, respect, altruism, respect for diversity, and maintenance of confidentiality; see the patient in a holistic perspective and give individualized care
		Work with respect for the universal human rights of women
		Demonstrate a commitment to excellence in all aspects of practice
		Work according to ethical standards and recognize and respond to ethical issues encountered in practice
		Recognize and manage conflicts of interest
		Exhibit professional behaviours in the use of technology-enabled communication
	Key competency 2: Demonstrate a commitment to society by recognizing and responding to societal expectations in health care	
		Demonstrate accountability to patients, society, and the profession by responding to societal expectations of physicians
	Key competency 3: Demonstrate a commitment to the profession by adhering to standards and participating in physician-led regulation	
		Fulfill and adhere to the professional and ethical codes, standards of practice, guidelines and laws governing practice
		Recognize and respond to unprofessional and unethical behaviours in physicians and other colleagues in the health care professions, according to the principles of effective feedback
		Participate in peer assessment and standard setting
	Key competency 4: Demonstrate a commitment to physician health and well-being to foster optimal patient care	
		Exhibit self-awareness and manage influences on personal well-being and professional performance
		Manage personal and professional demands, by maintaining a healthy work-life balance, for a sustainable practice throughout the physician life cycle
		Promote a culture that recognizes, supports, and responds effectively to colleagues in need by facilitating the process to help

Since two international validated frameworks were used to create the new framework, namely the CanMEDS framework and the UEMS framework, this framework should be considered as relevant in a more international context. Once it has been implemented in a new workplace-based learning curriculum in Belgium, it could form the basis of curriculum and assessment reform in other countries. The use of a postgraduate training curriculum, based on the same competency framework, that transcends national boundaries can enhance quality and transparency in medical education.

One of the limitations of this framework is that although only one competency did not reach consensus for relevance, multiple comments indicated that the competencies are too broad or difficult to assess in clinical practice. This weakness of the CanMEDS framework has already been acknowledged in earlier literature.^11^^, ^^19^ Moreover, the lack of appropriate assessment tools seems to be inherent in CBME.^2^^, ^^8^^, ^^19^^, ^^35^ It is important to limit the list of concrete learning outcomes when developing an assessment method. Otherwise, it may lead to ‘checkbox education’, where the holistic view of the original competencies is lost in an endless list of abilities.^3^^, ^^8^ Therefore, further investigation is needed to create an appropriate assessment tool to implement these competencies in clinical practice. One possibility would be the use of entrustable professional activities (EPAs) as an assessment method.^36^ EPAs are descriptors of work of which both the process and outcome can be assessed, it can be used as a way to translate enabling competencies into clinical practice. The competencies are achieved gradually in five levels of proficiency, in this manner all trainees have their own individualised learning curves and potential areas of concern could be addressed earlier.

The lists with specific gynaecological and obstetrical skills and knowledge added to the holistic competency framework were added as an example during this Delphi study but are not yet updated or validated. A subsequent investigation to optimise and validate these lists is required to create a complete picture of the postgraduate training in obstetrics and gynaecology.

## Conclusions

This article provides a new integrated competency framework for postgraduate training in obstetrics and gynaecology, based on three pre-existing frameworks. Consensus on this framework was reached through a Delphi study.  Such a holistic competency framework can form the basis of a curriculum reform in the postgraduate training in obstetrics and gynaecology within Belgium, but also in a more international context. Since a similar competency framework for paediatric postgraduate training also reached consensus in an earlier study, this competency framework could potentially serve as the basis for a curriculum reform of all specialist training programs in Belgium.

The competency framework in its current form is too extensive to use during workplace learning. Following steps should focus on optimization and validation of the specific gynaecological and obstetrical skills and knowledge, and on creating a qualitative assessment tool to integrate this competency framework into practice.

### Acknowledgements

The authors would like to acknowledge the contribution of Dr. Mieke Embo, Ms. Vasiliki Andreou, Ms. Oona Janssens and Ms. Sofie Van Ostaeyen for their expert opinions during the development of the competency framework. We would like to thank our participants for the investment of their time in this study.

### Conflicts of Interest

The authors declare they have no conflicts of interest.
